# University students’ experience of online space while engaging in synchronous learning *via* videoconferencing amidst the pandemic

**DOI:** 10.3389/fpsyg.2023.1083754

**Published:** 2023-01-30

**Authors:** Ahram Lee, Jee Young Lee, Eunju Jung

**Affiliations:** ^1^Deparment of Education, Sejong University, Seoul, Republic of Korea; ^2^Department of English Language and Literature, Korea University, Seoul, Republic of Korea; ^3^Graduate School of Education, Sejong University, Seoul, Republic of Korea

**Keywords:** online space, synchronous learning, videoconferencing, phenomenological approach, university students, COVID-19

## Abstract

**Background:**

The high infectivity and fatality of COVID-19 has changed the mode of higher education from onsite to online. Although many studies investigated the effectiveness and satisfaction of online education, little is known regarding university students’ lived experience of online space during synchronous learning *via* videoconferencing.

**Objective:**

The present study explored how university students experienced online space when engaging in synchronous learning *via* videoconferencing platforms during the outbreak of the pandemic.

**Method:**

The phenomenological approach was chosen to primarily explore students’ experience of online space as well as their experience of embodiment and relations to self and others. Interviews were conducted with nine university students who voluntarily participated to share their experience of online space.

**Results:**

Three core themes were generated from the descriptions of experiences provided by the participants. For each core theme, two sub-themes were emerged and described. The analysis of the themes demonstrated that online space was experienced as being separate from home but also inseparable because it was an extension of the comforts of home. This inseparableness is also reflected in the virtual classroom where the rectangular screen presented on the monitor is always shared with everyone in the class. Moreover, online space was perceived as having no transitional space in which spontaneity and new encounters occur. Finally, the presence of self and others was experienced differently in online space due to the participants’ choices of being seen or heard using their microphones and cameras. This led to a different sense of togetherness in online space. The insights gained from the study were discussed in relation to considerations for online learning in the post-pandemic era.

**Conclusion:**

Based on the emerged themes from the results, the current study concluded that the online space created by technologies cannot be a complete substitute for traditional face-to-face classrooms and suggested possible implications for designing and using online space in university education.

## 1. Introduction

“One of the most remarkable consequences of the closing of the gates was, indeed, a sudden separation of people who were not prepared for it… [they] found themselves abruptly and irremediably divided, prevented from meeting or communication with one another, because the gates were closed…” (Camus, 2021, pp. 77–78).

As Camus (2021) described in *The Plague* back in 1947, the closing of the gates after the declaration of the plague meant a total division of spaces and separation of the townspeople. Those within the closed gates could no longer see the faces of their loved ones beyond the gates. Their messages were sent in a 10-word dispatch that could barely deliver the essence of what they truly wanted to say. Closing of the gates meant complete separation.

Today, reflecting on the outbreak of COVID-19 which has affected the whole world, Camus’ illustration of the town of Oran back in the 1940s resonates with us ever so closely. Yet, one major aspect that strikes us differently is that even though the physical gates were closed, with social distancing policies and quarantine, we were still connected, seeing and hearing one another and making contact, in the online space.

The online space, created by the Internet, has embraced many parts of our lives amidst the pandemic. The technology was already on its way to rapid advancement, but the outbreak of the pandemic undoubtedly accelerated and expanded its use. One of the areas that experienced drastic changes due to the pandemic and the extended use of technology was the field of education. Schools around the world, including universities, were forced to close down their buildings and campus to prevent the spread of the contagion. Nevertheless, education continued. In many countries, most education sectors, from primary to higher education, took prompt action by replacing their traditional onsite learning with online learning (Aristovnik et al., 2020; Karasmanaki and Tsantopoulos, 2021; Yang and Huang, 2021). It was as though the whole campus had moved into the online realm.

Considering that onsite learning was not possible during the pandemic, online education was perceived as a useful alternative by both faculty and students (Almahasees et al., 2021). To better understand students’ online learning experiences, many studies compared synchronous and asynchronous learning; the former indicating real-time interactive learning *via* videoconferencing platforms and the latter referring to pre-recorded lectures accessible to learners at their convenience. For instance, Nguyen et al. (2021) found that undergraduate students preferred synchronous learning over asynchronous learning because it allowed them to be more engaged and motivated. Fabriz et al. (2021) also discovered that students who experienced more synchronous learning reported having more social interactions with peers as well as a sense of relatedness and overall satisfaction compared to those in an asynchronous learning setting. As demonstrated in these studies, synchronous learning seemed to provide a more engaging learning experience for learners. Universities in South Korea also expanded the implementation of videoconferencing platforms to continue courses in real-time and to increase interactions among students and instructors.

Videoconferencing tools, such as Zoom, Collaborate, and Webex, provide an excellent means for learning since they allow instructors and learners to have immediate interactions, not just through audio and video, but also through functions including chat rooms and interactive whiteboard (Martin et al., 2021). It should be noted, however, that studies comparing online interactive learning and face-to-face learning showed inconsistent results: Kunin et al. (2014) found that the face-to-face format was preferred over videoconferencing because technical factors, such as insecure Internet connection, affected the effectiveness and clarity of the presentation; on the other hand, Haney et al. (2012) reported that videoconferencing was perceived as effective as face-to-face lectures. In addition, factors such as the appropriateness of instructional design and course content (Kauffman, 2015) as well as learner characteristics including technology self-efficacy (Wang et al., 2013) were found to be related to online learning. While learners’ synchronous online learning experiences may be shaped by the use of technology, instructional methods, and learners’ characteristics, there is also the need to explore how students experience the online space in which learning takes place. Online learning *via* videoconferencing platforms entails various factors such as real-time interactions with peers and instructors, and yet, it may not be a complete substitute for face-to-face learning (Almahasees et al., 2021). This may be due to the fundamental differences between the online space and the onsite classroom.

Therefore, the purpose of the current study was to explore how university students experienced the online learning space when engaging in synchronous learning *via* videoconferencing platforms. Due to the pandemic, university campus life was inevitably transitioned to cyberspace, and students did not have any choice but to participate in online education. Hence, exploring students’ experience during the pandemic would yield different results from previous studies that investigated the experiences of students who made their own choice to take part in distance learning. Moreover, the lived experience of online space would guide educators and institutions as they continue to implement online education after the pandemic. Today, the world is focusing on recovery from COVID-19, and many aspects of our lives seem to return to the time before the pandemic. However, after experiencing the usefulness of videoconferencing tools, universities in South Korea continue to incorporate aspects of online learning. It is not a simple process for both instructors and learners to make the transition from face-to-face classroom experiences to an online learning environment (Henriksen et al., 2020), and if online learning *via* videoconferencing platforms were to continue, instructors would undoubtedly redesign instructions for online learning and institutions would provide stable online platforms. In addition to such efforts, understanding learners’ experience of the online space would provide deeper insight into the use of videoconferencing for learning as we move into the post-COVID-19 era.

Previous studies have examined the online space from a phenomenological perspective, primarily focusing on the *telepresence* along with embodiment and relations experienced within the space (Friesen, 2014, 2017; Berger, 2020). With these studies as a guide, the authors investigated how university students experienced the online space while they were engaging in courses delivered *via* videoconferencing platforms during the pandemic. Moreover, the current study explored how the experience of space was related to the experience of embodiment and relations to self and others.

## 2. Previous studies

### 2.1. Learning in online space

The term *online learning* was first used for WebCT developed in 1995 as the first Learning Management System (Singh and Thurman, 2019). Online learning has attracted many educators and students since it provides more flexible learning environments in terms of both time and place (Dhawan, 2020). Aligned with the advances in Information and Communications Technology (ICT), online learning has been employed to varying degrees in higher education, from a part of a lecture in a traditional face-to-face class to an institutional level such as Minerva University (2022), which is a private university headquartered in San Francisco, California, established in 2012 that employs multidimensional education through online seminars.

Online learning has various forms and a plethora of terms that are used as synonyms or definitions. In a recent systematic literature review on the definitions of online learning, Singh and Thurman (2019) found 46 definitions of online learning and synthesized them into two major forms focusing on the time element. The first form is asynchronous learning. In this learning environment, the instructor provides learning modules and interactive forums on an online platform, and students participate in the course activities at their own convenience. In asynchronous learning, students cannot get real-time support or feedback from their instructors or fellow students. The second form is synchronous learning in which teaching and learning as well as interactions among instructors and students occur in real-time. The advances in videoconferencing technologies allow this form of online education. In the synchronous online learning environment, students can receive immediate assistance and feedback from their instructor or classmates.

Previous studies have focused on comparing the effectiveness of face-to-face learning versus synchronous and/or asynchronous online learning. For instance, Martin et al. (2021) conducted a meta-analysis to analyze 19 studies that compared synchronous online learning to either face-to-face learning or asynchronous online learning at higher education levels in terms of cognitive or affective outcomes. They reported that a significant difference was only found between synchronous and asynchronous learning on cognitive outcomes, and it was speculated that students were more likely to be cognitively present in synchronous online learning than in asynchronous online learning (Martin et al., 2021). Almahasees et al. (2021) examined the perception of faculty and students on synchronous learning during the pandemic and found that, albeit useful, both faculty and students found it less effective compared to face-to-face learning. However, Francescucci and Rohani (2019) reported that even though there was no difference in students’ learning outcomes in an introductory marketing class between synchronous online learning and face-to-face learning, students in a synchronous learning class were less engaged than those in a face-to-face class. Considering the results of these studies, it may be concluded that although synchronous learning can be a convenient, effective, and interactive means of learning, it may not be a true replacement for onsite learning. Almahasees et al. (2021) also opined that online learning can only be a temporary substitute implemented in response to the drastic changes caused by the pandemic.

Learners’ experiences of online learning differ from those of face-to-face learning mainly due to a fundamental difference in the learning environment where learning takes place. Hence, previous studies on online learning have focused on the online learning environment that included factors such as the teacher’s teaching ability, curriculum design, and network support (Zhu et al., 2022), as well as the learning situation, learning activities, and teaching design (Pan, 2022). Specifically, Zhu et al. (2022) investigated the relationships among learning behavior, learning cognition, and learning environment in an online setting and found that the online learning environment positively impacted online learning behavior. Pan (2022) also examined the online environment as one of the factors that has a positive influence on learners’ empowerment. The quality of learning experiences in an online environment was also associated with external factors such as network services or access to mobile devices (Bast, 2021; Sarkar et al., 2021). However, only a few studies (e.g., Friesen, 2014, 2017; Berger, 2020) have examined the uniqueness of online space in which learners are connected with one another without physically being together. Therefore, empirical exploration is needed to deepen the understanding of learners’ experience of cyberspace.

While the online learning environment has been experienced by many distant learners for continuing education (Roddy et al., 2017; Lamon et al., 2020), the outbreak of COVID-19 has made online learning unavoidable to many university students. Learning in a university encompasses not just the cognitive elements that can be evaluated through exams but also social aspects. University is where students develop various generic competencies, such as interpersonal skills, that are applicable in a different context; and skills such as collaboration and communication were found to be associated with learning experiences that students identify as being significant to them (Lee and Lee, 2022). Martin et al. (2012) emphasized the importance of interaction in online classrooms and explored learners’ interaction with other learners and instructors, as well as with contents and interface in the virtual classroom; however, their primary focus was to discover ways to promote interactions. Since online space requires people to engage in different forms of communication and the presence of oneself and others are experienced differently (Friesen, 2014, 2017), how students experience online space and how the space affects their social interactions and relationships need to be examined.

### 2.2. Phenomenological approach

The current study implemented a phenomenological approach to explore students’ lived experience of online space while engaging in online courses *via* videoconferencing platforms during the pandemic. Phenomenology is defined as a methodological approach that aims to describe the core of a phenomenon told by the individuals who have experienced it (Teherani et al., 2015). Historically, phenomenology has been broadly categorized into two mainstreams: transcendental or descriptive phenomenology and hermeneutic or interpretive phenomenology depending on the chosen philosophical standpoints (Neubauer et al., 2019). First, transcendental phenomenology pursues objective descriptions of the crux of people’s lived experiences while emphasizing the epistemology of experience (What is an experience?); and it was asserted that objective description can be achieved by employing the method of “epoché” (also called bracketing) which precludes any judgment, predisposition, or interpretation of a researcher (Gill, 2020). Second, hermeneutic phenomenology focuses on the ontology of experiences (How does an experience exist?) and posits that interpretation is a necessary process of phenomenology (Gill, 2020). Accordingly, a researcher cannot perfectly bracket his/her presuppositions to make pure descriptions of the essence of a lived experience. Thus, hermeneutic phenomenology views a researcher as a method that provides a lens through which interpretations are made, and the current study has taken this position to explore the learners’ lived experience of online space.

The virtue of phenomenology is that it allows people to learn or gain insights from the detailed description of others’ lived experiences (Neubauer et al., 2019). According to the hermeneutic tradition of phenomenology, lived experiences can be interpreted through four existential dimensions: lived time, lived space, lived body, and lived human relations (Van Manen, 2016): lived time (temporality) means the personally and subjectively experienced time in our lifeworld; lived space (spatiality) implies how we think, feel, and construct the space in which we discover ourselves; lived body (corporeality) refers to how our body and mind experience everyday lives, including everything that is felt, unveiled, cloaked, and shared by our corporeal existence; and lived human relations (relationality) can be understood as the lived connections with others in our lifeworld. Although the four lifeworld existential entities can uniquely contribute to our understanding and interpretation of our lifeworld, more than one entity is interconnected to one another in the process of lifeworld interpretation (Rich et al., 2013). The primary focus of the current study was on spatiality, or university students’ lived experience of online space to be more specific, as well as corporeality and relationality in association with space.

## 3. Materials and methods

### 3.1. Research design

The current study was a qualitative study conducted through one-on-one interviews to explore students’ lived experience when engaging in synchronous learning via videoconferencing. As the lived experiences can be interpreted through lived time, space, body, and human relations (Van Manen, 2016), the authors implemented the phenomenological approach focusing on spatiality in relation to corporeality and relationality in order to capture students’ experience *via* videoconferencing platforms during the pandemic. Ethical approval was obtained from the Institutional Review Board of Sejong University, South Korea (SJU-2020-003 approved on December 2, 2020). The authors applied a criterion sampling strategy (Palys, 2008) to recruit participants who were willing to share their experience of online learning during the pandemic.

### 3.2. Participants

#### 3.2.1. Eligibility and recruitment

The participants of the study were university students who met the following criteria: (1) with experience of attending face-to-face classes on campus before the outbreak of COVID-19; (2) with experience of participating in online courses *via* videoconferencing platforms during the pandemic for over two semesters; and (3) with the willingness to verbally describe their experiences. Upon the ethical approval, a detailed research plan was posted on an online bulletin board of three elective courses at a 4-year university in Seoul, South Korea from December 7 to 18, 2020. Students who voluntarily wanted to participate in the study were asked to sign up for participation. The first author contacted each participant *via* phone to verbally explain the research aim and procedure before obtaining a written consent form *via* email.

#### 3.2.2. Sample characteristics

A total of nine students voluntarily participated in the research: six female and three male students. One student was in her fourth year, two were in their third year, and six were in their second year in university. The characteristics of the participants are provided in Table 1.

**Table 1:** Characteristics of the participants.

Participants	Gender	Year in University	Major
P1	Male	Third year	Industrial engineering
P2	Male	Second year	Business administration
P3	Female	Second year	International studies
P4	Female	Second year	German language and literature
P5	Female	Second year	Spanish language and literature
P6	Female	Third year	Business administration
P7	Female	Second year	Spanish language and literature
P8	Female	Fourth year	English language and literature
			Fashion convergence major
P9	Male	Second year	English language and literature

### 3.3. Data collection

An interview was conducted with each participant by the first author from December 29, 2020 to January 16, 2021 based on the open-ended questions generated by the authors. Due to the strict social distancing policy implemented in South Korea at the time, the interviews were conducted *via* phone or videoconferencing at a time convenient for the participants. Interviews lasted for 50–70 min and were audio recorded. Participants were first asked to freely describe their experiences of campus life and courses being transitioned to the online space during the pandemic, followed by probing questions based on their responses. After each interview, the recorded interviews were transcribed for analysis.

### 3.4. Analytic procedure

The collected data were analyzed by adopting a phenomenological approach. The process of analysis provided by Moustakas (1994) was followed. First, the authors read and reread the transcribed interviews and independently coded excerpts and expressions relevant to the experiences of the participants. Some of the examples of the initial codes included “convenience of online courses,” “difficulty of self-management,” “reduced sense of achievement after online activities,” “lack of communication,” and “feeling lethargic.” These initial codes generated by each author along with the relevant excerpts were then compared. For the texts that all the authors had identified and coded as relevant, the names of the codes were compared. During this process, redundant codes were combined or renamed, so that the selected codes may be used repeatedly for the detection of patterns (Saldaña, 2021). If some parts of the texts were coded by one author and not by the others, the authors went back to the original text to re-examine the context and determined whether the text was relevant to the study. Based on the consensus of the authors, the text was either coded or excluded.

Second, reduction and elimination processes were conducted. Vague expressions, insufficient descriptions, opinions, and expressions that were not related to the participants’ direct experiences were eliminated. For instance, the authors excluded codes that referred to participants’ perspectives or opinions on school policy mandating videoconferencing over recorded lectures or on the possible difficulty encountered by freshmen by attending online courses. Also, excerpts and codes that were irrelevant to online learning experiences, such as having a Zoom party with close friends, were eliminated.

Third, the remaining codes were clustered, and core themes and sub-themes were identified. The authors went back to the original text and the initial codes to ensure that the essence of the experiences was not lost. Since the experiences of online space and interactions were inevitably examined in comparison to the face-to-face encounters in real life, the authors focused on prepositional phrases, such as “on the screen,” “in the classroom,” “at home,” “sitting next to,” “side by side,” and “right in front of” when clustering the codes to draw the themes. The clustered themes were then labeled in more abstract terms to grasp the commonalities of the coded experiences, such as “omitted transitional spaces,” or to encompass the paradoxical state of the experiences, such as “separated yet inseparable spaces” and “present without presence.”

As advised by Saldaña (2021), the analysis was initially conducted in Korean, which was the language used in the interviews, in order not to lose any essential meaning from the interviews. When the final themes were identified, the authors translated the codes into English. The authors tried to avoid translating the codes word-for-word but instead searched for words, expressions, and idioms in order to accurately deliver the meaning. Then the relevant excerpts were selected and translated into English to be included in the manuscript. The excerpts were translated and back-translated to confirm that the intended meaning was accurately reflected in the translations.

## 4. Results

Through the analysis, 108 excerpts from the interview were coded, and three core themes were generated, each consisting of two sub-themes. The three core themes are *separated yet inseparable spaces*, *omitted transitional spaces*, and *present without presence*. The induced core themes and the constituting sub-themes are presented in Table 2.

**Table 2:** Core themes and constituting sub-themes.

Core themes	Sub-themes
Separated yet inseparable spaces	Joining the outer space from the comforts of home
	Sharing the unpartitionable online space
Omitted transitional spaces	Limited impromptu detours
	Roadblocks to crossing paths
Present without presence	See but not seen, hear but not heard
	Diluted meaning of togetherness

### 4.1. Separated yet inseparable spaces

The online space that you enter to attend a videoconferencing course is a space distinctly separate from the space taken up by your physical body, in which you are seated to look at the computer screen. However, since you are in two spaces — the online space where the course is being conducted and the space in which you are physically present—at the same time, the spaces are not experienced as completely separate.

Moreover, when you look at the virtual classroom space shown on the two-dimensional screen of a computer, each participant’s screen takes up a separate rectangular portion. Regardless of how vast the online space may be, what you actually experience is the two-dimensional screen displaying the faces of the participants, and the classroom space presented in front of you on the screen is shared by everyone looking into the screen. Although each rectangular border seems to divide the portion of the screen for each participant, the classroom space displayed on the screen is shared by everyone.

To encompass these phenomena, the authors named this theme *separated yet inseparable spaces*. The sub-themes include *joining outer space from the comforts of home* and *sharing the unpartitionable online space*.

#### 4.1.1. Joining the outer space from the comforts of home

In an ordinary face-to-face classroom, attending class means that you are physically in the classroom. You are either there or not. In an online videoconferencing class, however, you are in the online space attending the class, but at the same time physically elsewhere. Your body is at home, or in any other convenient place where you have access to the Internet and can enjoy private space to a certain extent, but you are also joined by others in the online space beyond your private space.

Bollnow (1961) explained that the lived space of humans fundamentally consists of inner and outer space; the former being the dwelling or the house, the secure and familiar spatial center of the life of an individual; and the latter being insecure, unknown, and hostile world yet full of wonders and charms. Being in the dwelling allows individuals to enjoy the comfort of familiarity and safety, yet people also have the craving to explore the wide, strange, and distant outer space. As Bollnow (1961) described, “Both security and danger belong to man, and consequently both areas of lived-space, as life develops in the tension between outer and inner space” (p. 34).

Such tension between outer and inner space is somewhat defused when students attend online videoconferencing classes because the outer space—the online classroom—is no longer so wide, strange, and distant since students are physically in their inner space. Students were still attending classes; they were in the virtual classroom, listening to lectures, participating in discussions and team projects, and interacting with fellow students, just as they would in a face-to-face classroom in outer space. Yet, physically being at home clearly meant that they were dwelling in their inner space.

Participants experienced videoconferencing courses as being “comfortable” and “convenient” because they were at home, but they also reported losing tension and concentration.

When I finished the first semester, I was getting a lot of stress because of the coronavirus, because the learning environment was, well, it would be different for each person, but students have a hard time concentrating on their studies at home, right? … I used to go out and study in the library or café but I could not do that anymore, so the exam was coming up but I could not concentrate… Overall, my satisfaction, understanding, and concentration in the courses dropped a lot… and I was really frustrated and stressed because of that. [P1]

… In my personal experience, um, it’s just, you cannot really get focused on studying alone at home… because I guess when you are home you feel so comfortable. And I noticed that on many occasions even though I did listen to certain lectures, I don’t remember much. No matter how effectively I take notes… In the last semester I decided to change my learning strategy so I would, you know, remember stuff better, and, um, so I tried to like get dressed, actually like put my makeup on and everything… so I would feel somehow awake and get the feeling that I’m going somewhere… It didn’t work. [P6]

In addition, participants reported that they lost the energy that they could have gained in outer space by physically going out and mingling with others.

I’m not a very, very outgoing person. I’m a person who likes to stay home, but even I felt somewhat saggy when I stayed home and didn’t go out for a few days… When I have something to do, I move around and become more efficient. But I have been taking classes at home, eating at home, and doing everything at home, and I feel that I am so much less efficient. I feel flabby and lethargic. That was the difficult part for me. [P3]

Bollnow (1961) stated, “Though the house is an area of security and peace for man, he would pine away if he locked himself in his house… his house would soon become a prison” (p. 34). By joining the online space to attend a course, students seem to move beyond the walls of their homes. Online space created with videoconferencing tools is where students go about their campus life; they can do things that they used to do in outer space, the actual classrooms. Nevertheless, with their body physically bound in their homes, they do not perceive online space as outer space, and the prolonged stay in the virtual space may even be experienced as a constraint.

#### 4.1.2. Sharing the unpartitionable online space

In the videoconferencing classroom, the space is always shared among all participants. That is, all the members are constantly shown on the two-dimensional screen facing forward. The participants are facing everyone on the screen, and even if you look at another person, in particular, the connection between the two people is not created on the screen. In a face-to-face classroom, the space is also shared by all participants, but in this three-dimensional space, you can physically sit next to someone, turn your head to make eye contact, and lean toward the other person to talk, creating a small, personal space between the two people within the big, public space of the classroom. In other words, sitting closer and leaning toward one another, making eye contact, and whispering can single someone out, partitioning a portion of the bigger space and creating your own temporary space separated by an invisible boundary. Such partitioning is not possible in the online space.

(Before COVID-19) I took language classes and back then we all helped and learned from one another. It used to be much easier, but now I’m doing it almost entirely alone. When I was taking the class face-to-face, even if I didn’t know the people around me at first, I got closer to them and got help from them during the class. You can’t do that in the non-face-to-face class. There is no such opportunity and you have to do things all alone which feels burdensome… When you are meeting people face-to-face, you can just ask, “What is it?” Just casually like that. But now you have to make intentional contact, send a cell phone message or something, and have to be determined to ask for help. That feels different from just casually asking a question face-to-face. [P5]

The videoconferencing tools allow the class to be divided into smaller rooms where group discussions can be conducted. Even so, the divided rooms are different from the partitioned space that you can experience in a face-to-face classroom. The division of the rooms is intentional and those who join a divided room are still sharing the room shown on the screen together. Such unpartitionable online space leads to a different type of communication to take place as the communication becomes purpose-driven in the online space (Friesen, 2014; Berger, 2020), where everyone is there for one reason—to take the course. Any other forms of personal talks or sharing of glances are restricted. There is no room for a quick chat about how difficult the assignment is, a quick peek at the classmate’s notes to check what you have just missed, or a quick tap on the shoulder of a person sitting next to you to ask ‘What did he just say?’

Without meeting face-to-face, being on the Internet is so, hmm, you get to talk only about stereotypical things. You end up talking about the topics of discussions and have fewer opportunities to talk about personal stuff. In that sense, I have less sense of belonging. [P8]

Why is it not easy to have a personal talk in the online classroom? The online space is shared by everyone equally. When you talk online, all attention is drawn to you. You cannot direct your speech to a certain person in the group. All is shared. In an actual classroom, leaning over and facing another person can initiate a conversation. As Pinchevski (2005) described, “the main characteristics of proximity is the realm wherein one can be affected by the other” (p. 218). Even in a public, open space such as a classroom, where several people are gathered, the proximity of faces can initiate private communication between the two people. It is this facing one another and leaning closer to the other person that creates proximity and room for personal interaction.

This relative distance and proximity among participants are only possible in a face-to-face classroom. In the online space, everyone equally shares a portion of a screen which seems to be clearly divided from others’ screens, yet the space is inseparable in that closer, more personal space between two people cannot be created.

### 4.2. Omitted transitional spaces

Casey (1993) described a “transitional space” as a realm that connects the inhabitation and the outside world.

A truly transitional space is often a place for creative action, providing enough protection to encourage experimentation (if not outright exploration) without being overly confining… Such a situation is like a reservation set aside so that certain actions not possible elsewhere can be undertaken here. (p. 122)

Friesen (2014) described “transitional spaces” as pathways, staircases, and elevators that reach the destination. For university students, transitional spaces are the hallways, staircases, and the space between the school buildings that they go through to reach the classroom. It is in these spaces where active interactions occur. However, in the online space, there are no transitional spaces. With a click of a mouse, you either enter or leave the online classroom. There is no rushing through the crowded hallway, stopping by the drinking fountain to get a sip of water, or standing side by side with a friend just outside the classroom chitchatting before the lecturer walks in. In the online space, transitional spaces are omitted. The sub-themes elaborating on this phenomenon are *limited impromptu detours* and *roadblocks to crossing paths*.

#### 4.2.1. Limited impromptu detours

On a university campus, casual interactions occur in transitional spaces that involve sharing personal issues, getting to know someone better, and gossiping about anything that goes around you. Online lectures exempt you from going through transitional spaces to arrive at a classroom. You enter the online classroom with a simple click, and you leave the classroom just as fast. Online space had no room for seemingly aimless and unstructured interactions.

As Participant 6 described, the campus was not just a place for learning but where her “life was basically revolving around” and that life was filled with spontaneous encounters occurring in the transitional spaces. Other participants also described casual, unintentional gatherings with friends, and sharing insignificant, trivial things which used to make life on campus fun and fruitful but are now lost because the courses are conducted online.

When I go to school, I often bump into my classmates, and after class, we grab dinner together and just talk. After class, we casually hang out. We go to random places or just sit on the lawn, freely talking about this and that. [P7]

My college life was like, bumping into friends in the plaza, grabbing snacks on the way, enjoying the weather, talking about things like this professor did this and that, that assignment is whatever, whatever, you know. But now, I don’t go to school. I attend classes by myself at home. It’s bed, class, and back to bed. I want to go back to the past because I miss the interactions. [P8]

Such small spontaneous enjoyments—having a good gossip and small chats, eating together, enjoying the weather, talking about school assignments, and just bumping into a classmate—shared with friends in the transitional spaces of the campus are deleted from students’ daily life when attending videoconferencing classes online.

#### 4.2.2. Roadblocks to crossing paths

Having no transitional spaces meant fewer opportunities for participants to meet new people on campus. There were team projects and group discussions through which participants interacted with others, but such interactions rarely developed into closer fellowship. As Participant 4 said, “you do not make friends in the classroom.”

Lampe et al. (2006) reported that even in social networking services (SNS), which are primarily designed for people to meet and socialize, people reinforce their friendships that were initiated offline rather than initiate new friendships. As Stutzman (2008) mentioned, online space is designed to reinforce pre-existing bonds. When this is so in social networking services, online classrooms make it even more difficult for new encounters.

I noticed that the freshmen are relying on *everytime*.*kr* because well, my friends and I can talk about stuff. How is this class, that class is annoying, what was the assignment, what is the test going to be about, I’m not going to class so sit in for me. We can talk about these things. But now, the freshmen can’t do that. They don’t have that community. They have to go to that anonymous online community to empathize with one another and solve problems. [P2]

At school… being around people, I got to expect some new encounters… now there is no such thing. In March and April, when the clubs are recruiting new members, I used to snoop around and listened to what they had to say, talked to random people, and enjoyed things like that. It’s just that such unexpected conversations, disappeared. Should I say unforeseen encounters and unforeseen conversations? Yeah. Those. [P9]

New encounters that used to occur in the transitional places helped participants build fellowship and communities of their own, which eventually helped them navigate through courses and other extracurricular activities. However, attending online videoconferencing courses meant reduced and limited opportunities to meet and interact with new people outside the classroom.

### 4.3. Present without presence

According to the Online Etymology Dictionary (n.d.), *presence* means the “fact of being present, state of being in a certain place and not some other.” When you are present in a place, it inevitably entails your body physically being there. People around you notice you are being there, and they can see and hear you. It is not your choice whether to be seen or heard, but just by being in a place, you are shown to others. In the online space, however, you have control over the use of the camera and the microphone, in most cases at least. You can be present in the online classroom, but others may not experience your presence because they may not see or hear you depending on the setting you use. Also, even if you may see other people on the screen, you do not *feel* them as you would in a face-to-face classroom. You are there in the classroom, but not really there. You are present without presence, which can be further illustrated by the following sub-themes: see but not seen, hear but not heard, and diluted meaning of togetherness.

#### 4.3.1. See but not seen, hear but not heard

Friesen (2017) stated, “In most social settings, to be seen by another is also to see him or her; to hear another is also (at least to have the possibility) to be heard; to touch is also to be felt (as ritualized in the handshake)” (p. 646). In a videoconferencing classroom, however, a student can choose to or is allowed to turn off the camera and the microphone depending on the setting of the class. When the size of the class is big, it is more likely that the instructor would mute all students and only have those making a remark turn their microphones on temporarily. When the class involves sharing a presentation material on the screen, only a limited number of participants are visible on the screen alongside the presentation material, tempting the audience to turn off their cameras. When the videoconferencing class is being recorded, the instructor cannot force students to keep their cameras on because of the issue of portrait rights. There may be many other reasons for students participating in a videoconferencing class to turn their cameras and microphones off. In such cases, students are present in the online classroom but are not actually seen or heard by other classmates or the instructor.

This semester, I had classes in the first, second, and third period, and they were all videoconferencing. The first period starts at 9 am… and I was always feeling tired and taking the course half-heartedly. I was there but not really there, but I had turned off the camera so the professor couldn’t have known. But of course, in the face-to-face classroom, the professor can see me so I would’ve been worried (about what she thought of me). [P4]

Especially in the case of Spanish major, we have this exam that we all prepare for… Originally, you would go to class and practice speaking with other people a lot so that you get more proficient in the language. But in the online classroom, people don’t turn on the camera and people tend to participate less or be more passive… I was grouped with people I met for the first time and we had to talk for like five minutes. I didn’t even know their faces but just had to say hello and talk only with my voice. It was a lot less effective than meeting and greeting people in person. [P7]

The participants’ experience resonated with the authors’ own experience as instructors, in that lecturing in front of a computer screen often felt like “talking to a wall” especially when it was difficult to see the students’ reactions because most of them had turned off their cameras. Online space is where you have the choice to be seen or heard. It liberates you from being poised the whole time during the class or paying attention to how you are being presented to others. Yet, it does affect how others experience your presence.

#### 4.3.2. Diluted meaning of togetherness

I’ve become a bit weary… taking courses together doesn’t make any sense now, right? So, my friend and I took the same courses together, but really, it wasn’t the same because we don’t go to the classroom together. So I just feel like my life is a bit impoverished. [P4]

To Participant 4, taking courses together meant going to the classroom together, sitting side by side, and working together. She was taking online courses together with her friend, and her friend was there on the screen but being side by side on a computer screen was not the same as sitting side by side.

Apparently, the non-face-to-face communication didn’t go so well, and it lacked the sense of them being there. As a result, my teammates and I naturally lost concentration, so, uh, I don’t think I was very satisfied with the quality (of communication). [P1]

Cambridge Dictionary (n.d.) defines the word *together* as “with each other.” According to this definition, you can be together with others in the online space. You are *with* your classmates and the instructor in the online videoconferencing classroom. Yet, participants reported that being in the online space together did not feel like being together. Participant 3 described that in the synchronous course, she was not learning Spanish directly from the professor because she was not facing the professor and exchanging glances.

Participant 9 also described how he lacked a sense of togetherness in his experience of videoconferencing courses, but he also mentioned how that experience was in part liberating for him.

In the case of Korean students, there is peer pressure that you receive from your classmates. I feel pressure when I try to ask questions in class. But that was reduced and I was rather free to ask questions… When I was participating in an online class, giving a presentation was a lot less burdensome because I thought that no one was actually watching me. Of course, there were people watching me, but they were not visually there in front of my eyes. [P9]

Participants 2, 4, and 8 also reported that they felt like “no one is looking anyway,” which made them feel lazy, less concerned about being prompt regarding their attendance, or pay less attention to their appearance. The authors also recalled the experience of lecturing to a larger group of students; the “whole class” would not fit in on one screen, and it was impossible to look around to see all the faces of the students at once. When a presentation material was being shared on the screen, fewer faces were displayed, making it even more difficult to scan through all the students in the classroom. As the participants described, the instructors were not fully aware of what they were doing. In sensing togetherness, it seems essential to physically be in the same space, make eye contact, and make connections with and pay attention to one another.

Lanier (2001) described how actual eye contact between participants of a videoconference is structurally impossible “because the camera and the display screen cannot be in the same spot… participants aren’t able to establish a sense of position relative to one another and therefore have no clear way to direct attention, approval or disapproval” (p. 68). When you look at the screen to see the faces of others, it inevitably prevents you from looking directly into the camera since the camera is not located at the center of the screen. Hence, others cannot directly feel that you are looking at them. This structural disparity caused by the positioning of the camera and the screen may lead to a reduced sense of togetherness in online space. You can see other people on the screen but you do not know what they are gazing at. The eyes never meet and you do not feel their presence in the way you would in a face-to-face classroom.

## 5. Discussion

With the prolonged outbreak of COVID-19 since early 2020, Korean universities adopted various online platforms to continue providing education to students in online space. Some courses were provided *via* recorded videos, but most were delivered in real-time *via* videoconferencing tools. Although videoconferencing can be an effective means to provide learning opportunities, the situation during the pandemic called for special attention because the students did not have the choice to choose whether to take certain courses online; it was as though their whole campus life was transitioned to the online realm. In order to reflect such an unprecedented context of inevitable engagement in online courses amidst the pandemic, the current study focused on the students’ experience of online space while taking courses *via* videoconferencing tools. The three core themes found in the study are *separated yet inseparable spaces*, *omitted transitional spaces*, and *present without presence*.

The first theme was *separated yet inseparable spaces*. Taking a videoconferencing course indicated that participants were in their dwelling while reaching the outer world at the same time. Even though participants were physically being in the comforts of their home, they perceived the online space as an inseparable extension of this comfort zone. Because the online classroom space was shared with everyone at all times regardless of the marked borders of each participant on the computer screen, it was not possible for the participants to lean toward someone to create proximity and initiate private conversations. Functions such as breakout rooms could relocate participants into smaller rooms and temporarily allow them to be disconnected from the main classroom, but participants still had no room for personal interactions. To the participants, the online space was experienced as a separate but not separable space.

The second theme was *omitted transitional spaces*. Transitional spaces, where opportunities to participate in various activities, have casual encounters, and build new relationships are possible, were non-existent in online spaces as going to and from the classroom happens with a simple click of a mouse. Participants recalled casual, spontaneous, aimless activities and conversations they had enjoyed in the face-to-face transitional spaces that were no longer fulfilled in the online space. Unintended surprises awaiting on the road to the classrooms were no longer available.

The third theme was *present without presence*. The participants were present, but they could not project their full presence to others in the online classrooms. In part, the sense of presence was reduced because participants could choose to turn their cameras and microphones off, restricting visual and auditory information that could communicate their presence to others. Also, even with the cameras and microphones on, participants expressed that the sense of togetherness was different in the online space as seeing others on the screen was not perceived as the same as sitting side by side with someone and doing things together. Participants reported that they felt as if no one was really looking at them.

Taking these themes into account, the current study found that students’ experience of online space cannot be explored apart from their experience of embodiment. Moreover, their experience of online space affected how they built and maintained relationships with others, and influenced their communication methods and content. By examining these experiences of university students who were attending online courses *via* videoconferencing platforms during the pandemic, the authors may gain insight into how to design and use online space for learning in the post-pandemic era. Even though the outbreak of COVID-19 had forced the full-scale implementation of online tools in the field of education, years of trial and error had stabilized online education. Wide applications of various technologies, such as videoconferencing platforms, have replicated the classroom experience in cyberspace (Turnbull et al., 2021). Students’ getting acquainted with online platforms and gaining competence in using digital tools have led to satisfaction with e-learning which in turn affected their academic achievement (Younas et al., 2022). Moreover, the use of educational apps during the pandemic has been found to affect the knowledge development of undergraduate students (Noor et al., 2022). Today, even after resuming face-to-face education, online learning is actively being discussed as a new option for university education. For instance, Noh et al. (2021) investigated the perceptions of university officials and relevant experts on the continued application of online education in universities and found that online education could become the new norm for universities in Korea. The discussions take a step further to examine the metaverse as a new platform to provide education (Kim et al., 2022). As the emphasis on online education continues even after the pandemic, the following practical implications are proposed based on the findings of the current study.

First, more attention should be drawn to the physical learning environment of learners when implementing online learning. As the findings of the current study illustrate, the physical location of the learners affected their experience in the virtual space. This finding is consistent with Ng (2021) conceptual model that attempted to explain the role of the physical environment in online distance learning. Various sensory stimuli and the presence of other people in the physical environment may affect learners’ cognitive and affective learning, as well as how learners engage in oral communication in the virtual classroom (Ng, 2021). That is, a physical environment that is not designed for learning may distract or prevent learners from turning on their microphones and cameras to participate in online activities. Almahasees et al. (2021) also found that students perceived their homes as not being suitable for attending online lectures due to external distractions. Although it would not be possible for all learners to control their learning environment, having open discussions about accessible learning environments or guiding students to design their personal space for learning may be beneficial.

Second, learners’ choices and expectations for online learning should be explored before implementing virtual classrooms in the post-pandemic era. Prior to the pandemic, studies on online learning were focusing on older adults with work commitments and family responsibilities who had made the choice to take online courses for flexibility and convenience (e.g., Roddy et al., 2017; Lamon et al., 2020). During the pandemic, however, general university students were unwillingly transferred into virtual classrooms. As the findings demonstrate, participants recognized and valued the convenience of online learning but still missed casual encounters occurring on campus, both in and outside the classrooms. Expecting that online learning will continue in certain educational sectors even after the pandemic, it should be considered that not all university students prioritize convenience and flexibility over on-campus interactions. In the study by Bai et al. (2022), it was found that college was a platform through which students access various resources and involve themselves in academics as well as social activities that affected learning outcomes. The facilities, resources, and activities accessible to students are offered not just in the classrooms but on the campus pathways. Hence, further study is needed to explore the significance of transitional spaces for university students.

Third, although there are technical restrictions to experiencing the sense of presence in virtual classrooms, various instructional methods and intentional efforts can be employed to enhance encounters among participants. Since prolonged participation in online learning may lead to videoconferencing fatigue which may in turn affect student engagement (Dacillo et al., 2022), students should have the option to manage their own cameras and microphones. Moreover, other ways to project one’s presence in online classrooms should be considered because making direct eye contact with a person on the screen is structurally impossible (Lanier, 2001). This is supported by Miao and Ma (2022) investigation as online interaction positively affected the way one projects oneself in the online space and builds relationships with others. Thus, increasing online interaction may lead to an enhanced experience of presence. Since interactions in videoconferencing spaces do not occur naturally and require more careful planning from lecturers (Koceski and Koceska, 2013), various instructional tools should be examined. For instance, Lamon et al. (2020) reported anonymous live polling as an effective function to have students contribute to class and feel a sense of belonging while not feeling embarrassed about getting an incorrect answer. Activity-based learning that motivates learners to pursue their own learning goals may also foster interactions among participants (Carr-Chellman and Duchastel, 2000). Using social media and group forums are other ways to foster communication among participants (Dhawan, 2020). Turnbull et al. (2021) also emphasized social media as a way to build a sense of “learning camaraderie” (p. 6414) in cyber classrooms.

These may be ways to improve learners’ experience of online learning, but the essence of online space does not change. As described in the current study, online space is inseparable from the experience of embodiment. Although dynamic and interactive online classrooms may be designed to enhance students’ engagement (Dhawan, 2020), students participating in the space are physically at home. These two synchronously joined spaces affect how students experience the online space. Moreover, students experience the online space on a two-dimensional screen where they do not experience relative proximity with their classmates or instructors.

This leads to a somewhat fundamental question of how university education and learning can be defined. If learning is considered as something occurring within the walls of a classroom, following well-designed instructions, the major focus can be on the effectiveness of online learning. However, if learning in a university is viewed as involving various competencies and skills that are developed through various forms of relationships and communications, online space naturally reduces certain opportunities as described in the results of the current study. Online space is experienced as distinct from a face-to-face classroom because it is accessed and presented on a two-dimensional screen, resulting in different perceptions of relatedness due to students’ physical absence in the shared space. In this regard, the question, “Do popular audio/visual technologies actually enable every day and educational communication that is “as good as” face-to-face?,” raised by Friesen (2017, p. 641) needs to be considered. Based on the results of the current study, the authors draw the conclusion that the online space created by technologies, albeit convenient and effective, cannot be a complete replacement for physical classrooms.

## Data availability statement

The raw data supporting the conclusions of this article will be made available by the authors, without undue reservation.

## Ethics statement

The studies involving human participants were reviewed and approved by Institutional Review Board, Sejong University. The patients/participants provided their written informed consent to participate in this study.

## Author contributions

AL and EJ conceptualized the study. AL conducted the interviews and acquired the funding. AL, JL, and EJ participated in the analysis and writing of the original draft. JL conducted the English editing. All authors contributed to the article and approved the submitted version.

## Funding

This research was partially supported by the funding for new faculty at Sejong University, Seoul, Republic of Korea, project number 20201114.

## Conflict of interest

The authors declare that the research was conducted in the absence of any commercial or financial relationships that could be construed as a potential conflict of interest.

## Publisher’s note

All claims expressed in this article are solely those of the authors and do not necessarily represent those of their affiliated organizations, or those of the publisher, the editors and the reviewers. Any product that may be evaluated in this article, or claim that may be made by its manufacturer, is not guaranteed or endorsed by the publisher.
